# Maxillary sinus haziness and facial swelling following suction drainage in the maxilla after orthognathic surgery

**DOI:** 10.1186/s40902-020-00277-0

**Published:** 2020-09-22

**Authors:** Jung-Soo Lee, Moon-Key Kim, Sang-Hoon Kang

**Affiliations:** 1 Department of Oral and Maxillofacial Surgery, National Health Insurance Service Ilsan Hospital, 100 Ilsan-ro, Ilsan-donggu, Goyang, Gyeonggi-do 10444 Republic of Korea; 2 Department of Oral and Maxillofacial Surgery, College of Dentistry, Yonsei University, 50-1 Yonsei-ro, Seodaemun-gu, Seoul, 03722 Republic of Korea

**Keywords:** Suction drainage, Orthognathic surgery, Maxillary sinus haziness, Facial swelling

## Abstract

**Background:**

We investigated the efficacy of a maxillary Jackson-Pratt (J-P) suction drain for preventing maxillary sinus hematoma and facial swelling after maxillary Le Fort I osteotomy (LF1).

**Methods:**

We retrospectively evaluated 66 patients who underwent LF1 at a single institution. Of these, 41 had a J-P suction tube inserted in the mandible and maxilla (maxillary insertion), and 25 had a J-P drain inserted in the mandible only (no maxillary insertion). Facial CT was obtained before and 4 days after surgery. We compared mean midfacial swelling and maxillary sinus haziness by *t* test and examined correlations between bleeding amount and body mass index (BMI).

**Results:**

For the maxillary-insertion group, the ratio of total maxillary sinus volume to haziness (57.5 ± 24.2%) was significantly lower than in the group without maxillary drain insertion (65.5% ± 20.3; *P* = .043). This latter group, however, did not have a significantly greater midfacial soft tissue volume (7575 mm^3^) than the maxillary-insertion group (7250 mm^3^; *P* = .728). BMI did not correlate significantly with bleeding amount or facial swelling.

**Conclusions:**

Suction drainage in the maxilla reduced maxillary sinus haziness after orthognathic surgery but did not significantly reduce midfacial swelling.

## Background

Postoperative complications of orthognathic surgery include bleeding, respiratory disorder, postoperative pain, soft tissue swelling, inflammatory reactions, infection, nausea, and vomiting [1]. During a Le Fort I osteotomy, bone cuts are made in the maxillary sinus, which can result in maxillary sinus changes and bleeding [2]. Blood accumulation and ischemia in the maxilla can lead to long-term inflammation [3].

The bleeding around the maxilla following a Le Fort I osteotomy needs to be reduced as much as possible to limit postoperative complications. An understanding of the anatomical structures related to the maxilla and a careful surgical approach can aid in achieving this goal. However, research is lacking on how to manage the maxillary sinus hematoma that can form after a maxillary osteotomy.

Postoperative midfacial swelling also needs to be considered after orthognathic surgery, which results in temporary swelling whether it is single or double jaw or performed on the maxilla or mandible. Swelling affects patient satisfaction with the aesthetic and functional outcome of orthognathic surgery [4]. Extensive research has targeted potential ways to reduce postoperative swelling, and basic methods include cooling the surgical anatomic area and administering corticosteroids [5, 6].

Primary swelling after orthognathic surgery is associated with blood accumulation at the surgical site [7], but what is unknown is how continuous blood drainage during the surgery might reduce postoperative blood accumulation and midfacial swelling. Suction drains are commonly used in orthognathic surgery and mandibular or maxillary osteotomies for this purpose, but their efficacy is unknown in the context of maxillary sinus hematoma and midfacial swelling. Because few studies have examined this question, we investigated the efficacy of a suction drain inserted in the maxilla for preventing postoperative hematoma of the maxillary sinus and facial swelling following a maxillary Le Fort I osteotomy during orthognathic surgery.

## Materials and methods

### Patients

We retrospectively evaluated patients who underwent orthognathic surgery at a single medical institution, including only those who underwent bimaxillary orthognathic surgery. Patients were excluded if they had undergone single jaw surgery of the maxilla or mandible, maxillary procedures other than Le Fort I osteotomy, mandibular procedures other than intraoral vertical ramus osteotomy and sagittal split ramus osteotomy, or additional osteotomies of the mandibular body or mandibular ramus. Radiographic haziness, including mucous retention cyst, was not frequently observed inside the maxillary sinus before orthognathic surgery. Patients with specified symptoms related to maxillary sinus pathology were excluded from this study.

Facial computed tomography (CT) (1 mm cut) scans displaying soft tissue from the forehead to the mandible were taken 1 month before orthognathic surgery. Three-dimensional (3D) simulation of orthognathic surgery was done on a computer before the actual surgery. Surgical wafers were generated based on simulation results from computer-aided design and computer-aided manufacturing. Height and weight were measured for each patient at the time of admission and used to calculate body mass index (BMI). Orthognathic surgery was performed on both the mandible and maxilla, with Le Fort I osteotomy of the maxilla being performed on all patients. Jackson-Pratt (J-P) suction tubes were bilaterally inserted with respect to the site of the mandibular ramal osteotomy to drain blood. Patients were excluded if negative pressure was not generated in the suction drain, the tube fell out within 2 days, postoperative CT scans 4 days after surgery were unavailable, or CT slice thickness exceeded 1 mm. In total, 66 patients with or without a J-P suction tube in the maxilla were included.

### Drain insertion following the maxillary Le fort I osteotomy

Patients were divided into two groups according to whether a suction tube was inserted during the maxillary Le Fort I osteotomy. Of these 66 patients who underwent LF1 at a single institution, 41 had a J-P suction tube inserted in the mandible and maxilla (maxillary insertion), and 25 had a J-P drain inserted in the mandible only (no maxillary insertion).

**Fig. 1:**
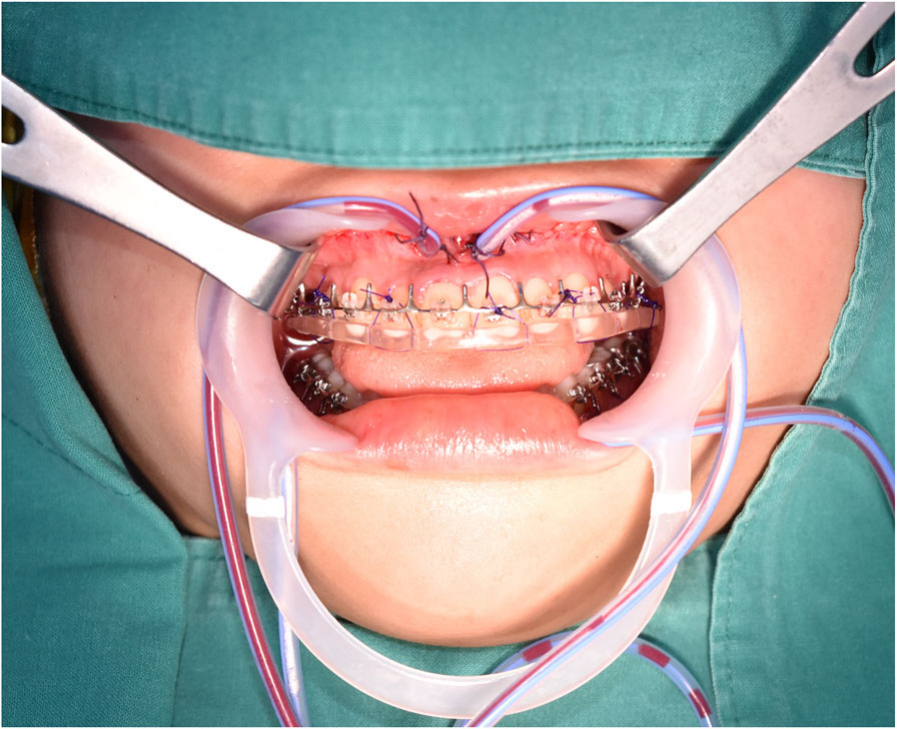
The drainage tube inserted on the maxillary Le Fort I osteotomy and anchored to the maxillary labial frenum

Insertion was accomplished as follows. A mucoperiosteum was bilaterally punctured with respect to the labial frenum in the anterior maxilla and into the periosteum using the needle portion of the J-P drain after the maxillary Le Fort I osteotomy. After the needle was separated from the drainage tube and used for making punctures, the nail was inverted to insert the part connecting the needle and the drainage tube into the periosteum through the puncture. Once the needle and drainage tube were reconnected underneath the periosteum, the tube was passed through the puncture to the outside of the periosteum so that the drain tube with holes was located to the maxillary Le Fort I osteotomy site and on the posterior cutting plane of the maxilla. The drainage tube was sutured to the maxillary labial frenum, and primary closure was performed at the incision site (Fig. 1). A silicone suction reservoir (100 mL) was connected to the tube, and blood was drained by application of negative pressure.

### Postoperative care

After surgery, 125 mg of methylprednisolone was intravenously administered. All patients were admitted to the intensive care unit for airway management immediately after the surgery and transferred to the general ward within 2 days after recovering satisfactorily. Patients were intravenously administered a non-steroidal anti-inflammatory drug, ketorolac, for postoperative pain management unless they displayed unusual symptoms.

The volume of blood drained through the suction tube in the maxilla and mandible was measured for 2 days after the surgery. Suction blood volume of J-P drain in both the mandibular and maxillary areas (*n* = 41) is the sum of drain volume in maxillary and the mandibular area. Negative pressure was continuously applied to induce drainage for this period. Cases were excluded if negative pressure could not be generated in the J-P suction tube because of air leakage. Suction tubes were removed 2 days after surgery. Facial CT scans (1 mm cut) were obtained 4 days after surgery.

### Postoperative 3D evaluation of the maxillary sinus with or without J-P suction tube insertion

**Fig. 2:**
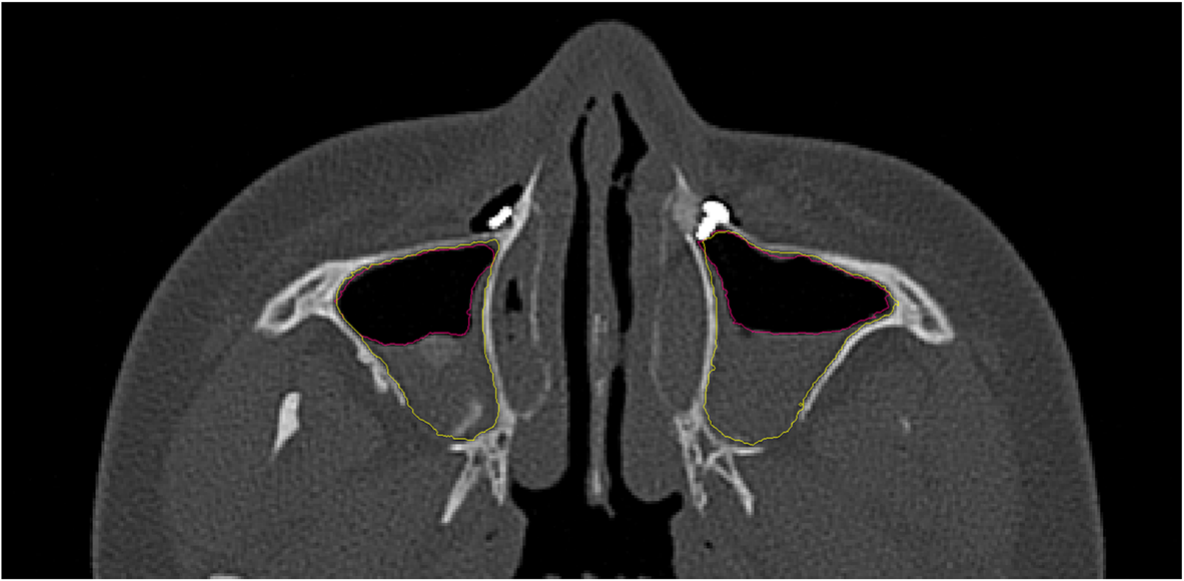
Borders of the empty spaces (red) within the maxillary sinus (yellow) according to the white gray values were identified on all CT scans at the level of the maxillary sinus

We compared the amount of maxillary sinus hematoma and midfacial swelling between the group that had maxillary J-P suction tube insertion and the group that did not, using facial CT scans obtained 4 days after surgery. To measure the volume of blood accumulation in the maxillary sinus, we used the haziness around the maxillary sinus on postoperative facial CT. We also measured the total maxillary sinus volume and air level volume showing vacancy of maxillary sinus on postoperative CT. The borders of the empty spaces within the maxillary sinus according to the white gray values were identified on all CT scans at the level of the maxillary sinus (Fig. 2). Because the size of the maxillary sinus varies, we compared ratios between total maxillary sinus volume and the volume showing haziness on CT between patients with a J-P suction tube and those without. For this 3D volume comparison, we used Mimics (14 version, Materialize, Leuven, Belgium) software and calculated separate ratios for the right and left maxillary sinuses.

**Fig. 3:**
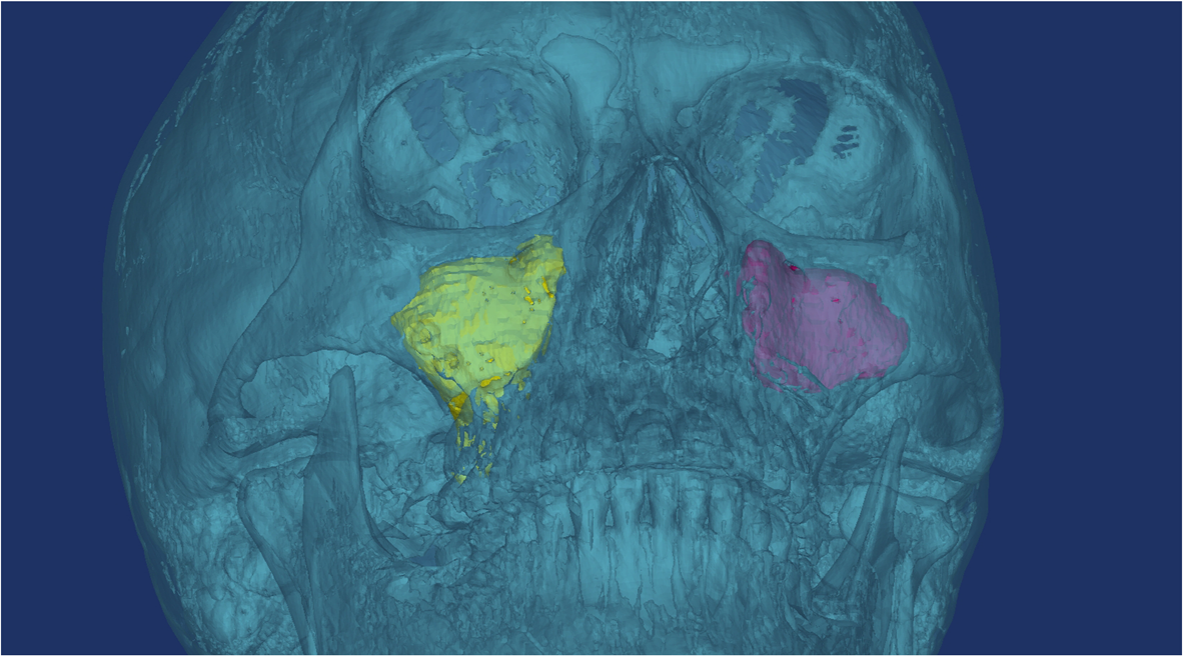
3D reconstruction of the maxillary sinus showing haziness, determined by subtracting the empty space (e.g., red left maxilla) volume of the maxillary sinus from the total maxillary sinus volume (e.g., yellow in the right maxilla) in each maxillary sinus after surgery

To delineate the area for volume measurement, we identified the borders of the maxillary sinus to mark its scope and created 3D reconstructions of the CT slices to measure the volume. We calculated the volume showing haziness (indicating accumulated blood) by subtracting the volume of empty space within the maxillary sinus from the total volume after surgery (Fig. 3). After making these volume measurements for the right and left sides, we used them to calculate the ratio between total maxillary sinus volume and volume of accumulated blood and compared them between patients with and without a maxillary J-P tube.

### Postoperative 3D evaluation of facial swelling with and without J-P suction tube insertion

Facial CT scans obtained within 1 month before and 4 days after surgery were used to compare midfacial swelling between patients with and without a J-P suction tube inserted in the maxilla. To measure the amount of midfacial swelling after a Le Fort I osteotomy, we used differences in facial volumes. For this purpose, we 3D reconstructed the Digital Imaging and Communications in Medicine (i.e., DICOM) files of the preoperative and postoperative facial CT scans, using Mimics software, and exported them as stereolithography files. These files were subjected to surface-based registration with respect to the cranium and imported back to Mimics.

**Fig. 4:**
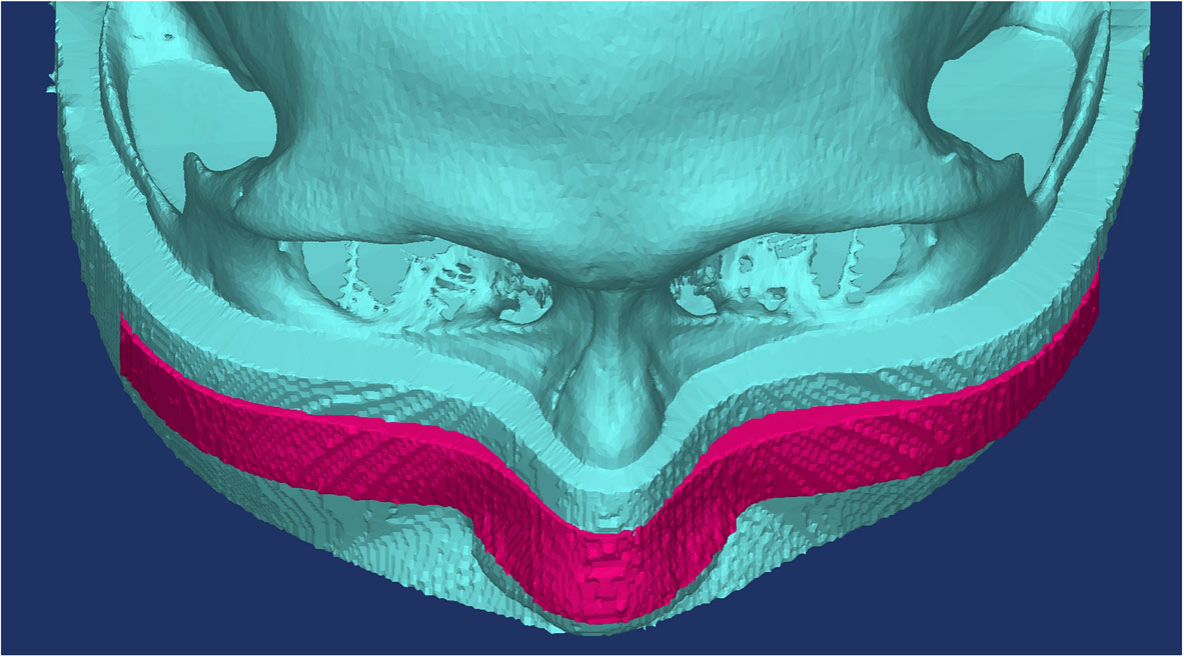
Differences (red) in facial volumes between preoperative (blue) and postoperative facial 3D-reconstructed CT data used to measure the amount of midfacial swelling with a 3D model after a Le Fort I osteotomy

We examined the soft tissue above the site of the maxillary Le Fort I osteotomy and 15 mm below the surface parallel to the FrankFort horizontal plane and passing by the infraorbital foramen. This soft tissue was on a 140-mm-wide sagittal plane lying perpendicular to the horizontal plane and passing by the furthest lateral point on the zygomatic arch. Differences in the facial volume before and after surgery were calculated as a measure of facial swelling using a 3D model (Fig. 4). We first determined the area of soft tissue to be examined for assessment of swelling on the postoperative CT. After overlapping the preoperative CT scans and 3D images of the skull, we inferred facial swelling from the volume of remaining soft tissue after taking the difference between preoperative and postoperative scans.

### Statistical methods

We used the *t* test to compare the mean amount of midfacial swelling and haziness in the maxillary sinus. We also compared mean age and BMI between the groups with and without a maxillary J-P suction tube and examined the correlation between the amount of bleeding and BMI in each group. For statistical analyses, we used IBM SPSS Statistics version 23 (IBM Corp., Armonk, NY, USA) and set *P* < .05 to indicate statistical significance.

## Results

As noted, 66 patients were included, 41 who had a J-P suction tube inserted in both the mandible and maxilla, and 25 who had a J-P suction drain inserted in the mandible only (no maxillary suction drain). The sex ratio was 18:23 (female:male) and mean age (±SD) was 22.2 ± 3.5 years in the group with a maxillary suction tube, compared to 11:14 and 23.8 ± 6.3 years in the group without a maxillary suction tube.

The mean BMI (±SD) of the 41 patients with a maxillary J-P suction tube was 22.8 ± 3.3 kg/m^2^, and the mean (±SD) amount of blood drained in the 2 days after surgery was 224.8 ± 102.0 mL. Suction blood volume of J-P drain in both the mandibular and maxillary areas (*n* = 41) is the sum of drain volume in maxillary and the mandibular area. The mean BMI (±SD) of the 25 patients without a maxillary J-P suction tube was 22.8 ± 3.3 kg/m^2^ (*P* = .970 vs other group), and in the 2 days after surgery, they had a mean (±SD) 144.4 ± 67.9 mL of blood drained (*P* < .001 vs other group).

We also examined postoperative changes in the 3D models of the maxillary sinus in patients with and without a maxillary J-P suction tube. The mean total maxillary sinus volume (±SD) was 21,441 ± 6239 for the left and right maxillary sinuses of the 41 patients with a maxillary suction tube, and their mean (±SD) haziness volume was 11,995 ± 5510 mm^3^. In this group with a maxillary sinus suction tube, the average ratio (±SD) between total maxillary sinus volume and haziness volume was 57.5% ± 24.2%.

Among the 25 patients with no maxillary J-P suction tube, the mean (±SD) total maxillary sinus volume was 18,756 ± 5726 mm^3^ for the left and right maxillary sinuses. The mean (±SD) maxillary sinus haziness volume was 12,082 ± 5028 mm^3^, and the average (±SD) ratio between the total maxillary sinus volume and haziness volume was 65.5% ± 20.3%. This ratio was significantly higher than that observed in the group with a maxillary J-P suction tube (*P* = .043) (Table 1).

**Table 1:** Results of maxillary sinus haziness and facial swelling following suction drainage in the maxilla according to the drain insertion after orthognathic surgery

	Orthognathic surgery patients (*n* = 66)		
Variables	J-P drain in both the mandibular and maxillary areas (*n* = 41)	J-P drain insertion only in the mandibular area (*n* = 25)	*P*
Sex (male:female)	23:18	14:11	.597
Age (mean ± SD) years	22.2 ± 3.5	23.8 ± 6.3	.274
Suction blood volume (mean ± SD) (mL)	224.8 ± 102.0	144.4 ± 67.9	< .001*
BMI (kg/m^2^)	22.8 ± 3.3	22.8 ± 3.3	.97
Maxillary haziness (mean ± SD) (%)	57.5 ± 24.2	65.5 ± 20.3	.043*
Facial swelling volume (mean ± SD) (mm^3^)	7250 ± 3483	7575 ± 3942	.728

*Significant: *P* < 0.05

†*Abbreviation*: *J-P* Jackson-Pratt (J-P) suction tube, *BMI* body mass index

Suction blood volume; blood volume using J-P drain including mandibular J-P drain during 2 postoperative days

Using the unpaired Student’s *t* test, we compared the age, BMI, maxillary haziness, and facial swelling volume between the two groups

Suction blood volume of J-P drain in both the mandibular and maxillary areas (*n* = 41): sum of drain volume in maxillary and the mandibular area

We also examined changes in facial swelling following the insertion of the J-P suction tube. The mean volume (±SD) of the midfacial soft tissue was 7250 ± 3483 mm^3^ in the group with maxillary insertion of the tube, compared to a mean (±SD) volume of 7575 ± 3942 mm^3^ for those without maxillary tube insertion (not significant; *P* = .728).

BMI did not significantly correlate with the amount of bleeding in the group with maxillary tube insertion (*P* = .260) or the group without it (*P* = .149). Neither group showed a correlation between BMI and facial swelling, either (*P* = .744 with maxillary tube; *P* = .359 without).

## Discussion

In this study, we compared the ratio of total maxillary sinus volume and maxillary sinus hematoma volume to examine the effect of J-P suction drainage following a maxillary Le Fort I osteotomy. This ratio was 57.5% for the group with maxillary J-P suction tube insertion and 65.5% for the group without maxillary tube insertion, for a statistically significant difference between the two groups (*P* = .043).

The bones around the maxillary sinus become separated following a Le Fort I osteotomy, leading to blood accumulation and mucosal thickening in the maxillary sinus [8]. Blood accumulation in this region prolongs mucosal thickening and can cause maxillary sinusitis as a complication of this procedure [9, 10]. The incidence of complications associated with maxillary sinusitis after a maxillary osteotomy is reported to be 1.1% [3], with a prevalence of 4.76% [11]. In this study, 41 patients received a J-P suction tube inserted at the site of the maxillary Le Fort I osteotomy to drain blood continuously for 2 days after surgery and reduce maxillary sinus haziness on CT. Based on the results of this study, the use of a suction drain in the maxilla after orthognathic surgery is recommended.

Extensive research has been conducted on orthognathic surgery and maxillary sinusitis [3, 11–13] but has left a gap regarding methods for reducing maxillary sinus haziness on CT after maxillary Le Fort I osteotomy. One group reported that reduced operative time and antibiotics could help reduce mucosal thickening after a maxillary osteotomy [8]. Others have used the Lund–Mackay score to measure inflammation in the maxillary sinus [9, 13]. Here, we used quantitative methods to evaluate postoperative maxillary sinus and haziness volumes using 3D reconstructions of 1 mm CT slices. We found that use of a maxillary suction drain following this surgery reduces maxillary sinus haziness on CT.

Maxillary sinus-related postoperative complications after an orthognathic surgery have been previously reported. It is crucial to reduce the amount of persistent hematoma and effusion in the maxillary sinus after a surgical operation. Along with complications involving the maxillary sinus, persistent hematoma and effusion can also be linked to other symptoms caused by drainage of blood into the nasal cavity, thus causing nasal congestion, dyspnea, and non-mucous membrane swelling. Moreover, limiting persistent hematoma and effusion can assist in efficient postoperative management of the sectioned maxilla and the adjacent maxillary sinus. The involvement of the maxillary sinus could be related to other indirect complications, such as rhinitis and empyema. Therefore, radiographic haziness of the maxillary sinus can be an essential parameter in research and clinical treatment related to orthognathic surgery.

A mean maxillary sinus volume of 33.44 cm^3^ has been reported following orthognathic surgery of Class III malocclusions [14]. However, the maxillary sinus volume can vary depending on the procedure performed and variations in human skeletal size. Mean volumes from 18.86 cm^3^ to 32.05 cm^3^ have been reported, along with variations among ethnic groups [15]. The maxillary sinus volume also can be changed after orthognathic surgery. In this study, the mean postoperative maxillary sinus volume was around 20.10 cm^3^.

A Le Fort I osteotomy can lead to midfacial swelling because of bleeding around the maxillary sinus after maxillary advancement or retraction. Temporary swelling also can occur around the philtrum below the nose and in the suborbital region. Various methods have been used to assess facial soft tissue swelling after orthognathic surgery [4, 5, 16, 17], including 3D techniques involving CT and lasers and ultrasonography to measure soft tissue thickness [4, 5, 16–18]. A standardized method to assess facial swelling is lacking, however. Severe facial swelling occurs after the orthognathic surgery. However, no statistical difference in the volumes of edema was observed between the group with an inserted JP suction tube versus the group without an inserted JP suction tube, though the group without a suction tube had a higher value. In this study, the number of patients was 41 and 25, respectively. In a similar study in the future, with an appropriately larger sample size, statistically significant findings can be expected.

Here, we measured the volume of soft tissue starting from the infraorbital foramen to a point 15 mm below it to eliminate the influence of maxillary movement after a Le Fort I osteotomy and variations in facial widths. A disadvantage of our approach is its limited efficacy in assessing swelling in the whole face. Patients had different soft tissue widths because of different zygomatic widths (minimum, 112.6 mm–maximum, 143.9 mm). To reduce errors caused by the width difference, the soft tissue width was set to 140 mm for all patients. The height of the soft tissue was measured at 15 mm for all patients. With this approach, we found no significant differences in soft tissue volume between patients who had a maxillary J-P suction tube inserted (7250 mm^3^) and those without a maxillary tube (7575 mm^3^; *P* = .728).

To evaluate maxillary sinus involvement (radiographic haziness) after orthognathic surgery, we reconstructed the sinus into a three-dimensional image using the pre-surgical CT scan images and measured its volume. We considered bilateral maxillary sinuses of each patient. After the surgery, we reconstructed and measured the volume of the haziness in the sinus. The postsurgical findings of the sinus varied for each side and for each patient. Therefore, for each sinus, the volume of the haziness was divided by the volume of the maxillary sinus and converted as percentage. Using this proportion, we conducted the Student *t* test for comparative evaluation and statistical significance between the two groups, that is, groups with and without an inserted tube.

With respect to the facial swelling analysis, the volume of the post-surgical enlarged soft tissue was measured using the three-dimensional reconstructed CT images. We had assessed the preoperative facial tissue using the same method. We then superimposed the pre- and post-surgery reconstructed images using the cranial portion as reference, as it remains unaltered after the orthognathic surgery. In the superimposed volume, we subtracted the preoperative tissue, and could thus obtain the measurements of the enlarged tissue only. Using the unpaired Student’s *t* test, we compared the soft tissue enlargement between the two groups.

Since each patient shows varied facial dimensions, the horizontal and vertical lengths were determined, followed by measurement of the differences in the soft tissue within these determined areas.

We also found no correlation of BMI with swelling, although one group has reported a non-significant correlation of increased BMI with swelling after orthognathic surgery [4]. In the current study, all patients were intravenously administered 125 mg of methylprednisolone after orthognathic surgery and facial cooling treatment for 2 days. Postoperative administration of corticosteroids has been proven to reduce swelling effectively [19]. Facial cooling is also a basic method to reduce postoperative swelling [20]. Further research is needed to develop more effective methods to reduce swelling after orthognathic surgery.

BMI also did not correlate significantly with the amount of bleeding in our patients. Other groups have investigated factors associated with bleeding in the maxillary sinus after orthognathic surgery [18, 21]. One study showed that lower body weights are associated with lower hematologic parameter measurements and that bleeding increases with operative time, decreases with increasing body weight, and does not significantly correlate with age [21]. However, few studies have examined maxillary sinus changes following orthognathic surgery, and further research is needed on management with different BMIs, drain types, and methods of drain insertion.

In this study, the mean volume of blood drained through the J-P drain for 2 days after surgery was 144 mL for the 25 patients who had a J-P suction tube inserted in the mandible and 224 mL for the 41 patients who had a J-P suction tube inserted in the mandible and maxilla. The volume of blood drained was not separately measured for the maxilla and mandible for the patients who had a J-P drainage tube inserted in both the maxilla and mandible. This is also drawback of the current study. However, based on the differences in the mean volume of blood drained between the two groups, the volume of blood drained in the maxilla was estimated to be 80 mL.

Several types of drains available include gauze, rubber, and suction drains, and suction drains are commonly used in oral and maxillofacial treatments. Draining blood and reducing blood accumulation in the maxillary sinus following maxillary osteotomy requires continuously generated negative pressure via a J-P suction tube. However, air leakage may occur at the site of tube insertion within 2 postoperative days, leading to loss of the negative pressure to be lost and blocking effective draining from the maxillary sinus. The suction tube must be carefully inserted and managed.

A limitation of this study is that because we used CT scans obtained 4 days after orthognathic surgery, we could not assess changes arising before that time point. The effect of the J-P suction drain could have been underestimated, given that facial swelling is severe during the first few days after surgery. In addition, this was a short-term observational study using the CT scans of a limited number of patients. A long-term study with a larger patient population is needed for an accurate assessment of the efficacy of drainage suction tubes inserted in the maxilla. Future research should aim at developing and improving methods of evaluating hematoma within the maxillary sinus and measuring postoperative facial swelling.

Unfortunately, our study did not consider the direction and the amount of movement for the Le Fort I maxillary fragment. In this study, there was no horizontal advancement of the maxilla. However, the posterior maxillary impaction may have caused forward tilt. Therefore, to minimize the variation caused by this change, the edema in the region superior to the Le Fort I osteotomy site was measured.

However, restricting the measurement areas to avoid the effects of surgery and to measure facial swelling solely may result in inaccurate dimensions, which may be a limitation of this study. In my opinion, a more reliable outcome is possible if the direction and amount of movement of maxilla is considered during the facial swelling analysis. Few studies have compared post-surgical facial swelling and maxillary sinus radiographic haziness with the direction and amount of the movement of the maxilla. Advanced related studies are recommended in the future.

## Conclusions

The use of a suction drain did not lead to a statistically significant reduction in facial swelling after orthognathic surgery. However, suction drainage in the maxilla reduced haziness on postoperative CT scans of the maxillary sinus. Insertion of a J-P suction tube in the maxilla may be considered a routine procedure following a Le Fort I osteotomy of the maxilla.

## Data Availability

The datasets used and/or analyzed during the current study are available from the corresponding author upon reasonable request.

## Abbreviations

LF1: Le Fort I osteotomy
CT: Computed tomography
3D: Three-dimensional
BMI: Body mass index
J-P: Jackson-Pratt
DICOM: Digital Imaging and Communications in Medicine
SD: Standard deviation
